# A risk-adapted, dose-optimized esophagus-sparing radiation therapy technique for high-risk patients with lung cancer: feasibility, dosimetry, and acute esophageal toxicity

**DOI:** 10.1186/s13014-026-02880-3

**Published:** 2026-06-23

**Authors:** Jia-Qi Huang, Shu-Yan Li, Huan Li, Jing-Jing Cao, Jia-Yi Chen, Wei-Xiang Qi, Sheng-Guang Zhao

**Affiliations:** 1 Department of Radiation Oncology, Ruijin Hospital, Shanghai Jiao Tong University School of Medicine, Shanghai, 200025 China; 2 Shanghai Key Laboratory of Proton Therapy, Shanghai, 201801 China

**Keywords:** Acute radiation-induced esophagitis, Esophagus sparing, Dose optimization, Thoracic radiation therapy, Lung cancer

## Abstract

**Background:**

Acute radiation-induced esophagitis (ARIE) remains a clinically significant toxicity during definitive thoracic radiation therapy (TRT), particularly in patients with close esophagus–planning target volume (PTV) proximity. This study evaluated a risk-adapted, dose-optimized esophagus-sparing (ES) strategy designed to reduce ARIE while maintaining adequate target coverage in high-risk patients.

**Methods:**

Eighty patients with lung cancer treated with definitive TRT between May 2023 and January 2025 were enrolled. The injury-effective esophagus (IEE) was defined as the local high-risk esophageal segment consisting of the central-IEE and an additional 2-cm superior and inferior cranio-caudal expansion. The central-IEE encompassed all axial levels where PTV–esophagus overlap was present, and the ES region was contoured as the portion of the IEE outside the PTV. Based on the degree of PTV/central-IEE overlap (< 1/3, 1/3–2/3, > 2/3), the ES mean dose was constrained to 1/3, 1/2, and 2/3 of the prescribed dose, respectively. ARIE was graded using Radiation Therapy Oncology Group (RTOG) criteria. Progression-free survival (PFS) was defined from initiation of the TRT to progression or death; overall survival (OS) was also evaluated.

**Results:**

Among 80 high-risk patients with the esophagus located within 5 mm of the clinical target volume (CTV), ARIE occurred in 24 patients (30.0%), including only one Grade 3 event and no Grade ≥ 4 toxicity. For contextual reference, the incidence of any-grade ARIE in a pre-ES historical benchmark cohort was 76.5%. In a paired dosimetric comparison of 10 cases, the ES strategy reduced esophageal dose exposure, particularly within the ES region, while substantially preserving target coverage and maintaining acceptable organ at risk (OAR) constraints. Exploratory univariable analyses suggested associations between ARIE and concurrent chemo-immuno-radiation therapy (CIRT), the geometric extent of the ES region, higher esophageal dose exposure, and failure to pass the ES-plan. Survival outcomes were descriptive only.

**Conclusions:**

This risk-adapted, dose-optimized segmental ES strategy appears feasible and may reduce clinically relevant ARIE with broadly preserved target coverage. Prospective validation is warranted.

**Supplementary Information:**

The online version contains supplementary material available at https://doi.org/10.1186/s13014-026-02880-3.

## Introduction

Thoracic radiation therapy (TRT) remains a cornerstone in the multidisciplinary management of lung cancer. Acute radiation-induced esophagitis (ARIE) is a common dose-limiting toxicity that may impair quality of life, necessitate treatment interruption, and restrict thoracic dose escalation [1–5]. Among patients with non-small cell lung cancer (NSCLC) undergoing standard definitive concurrent chemo-radiation therapy (dCCRT), the reported incidence of severe esophagitis ranges from 7% to 23% [1, 6–8]. Conversely, patients with small cell lung cancer (SCLC) may experience higher rates, with reported incidences between 13% and 31% [9–12]. Consequently, mitigation of ARIE represents a persistent challenge in thoracic radiation planning.

To reduce ARIE risk, esophagus-sparing (ES) strategies have increasingly been incorporated into treatment planning, particularly for high-risk patients. Prior studies have identified several clinical and dosimetric predictors of ARIE, among which the mean dose to the esophagus consistently demonstrates a dose-toxicity relationship [6, 13, 14]. Existing approaches typically focus on reducing dose gradients outside the planning target volume (PTV) [15–20] or selectively sparing the contralateral esophagus [21–23]. However, these strategies generally treat the esophagus as a homogeneous organ at risk (OAR) and may not account for spatial heterogeneity in dose exposure relative to target geometry. In clinical practice, some esophageal segments may lie outside high-dose regions and therefore may not require active sparing, highlighting the need for more region-specific planning approaches.

In this study, we developed a risk-adapted, region-specific ES strategy that targets the contralateral portion of the injury-effective esophagus (IEE) adjacent to the PTV. We hypothesized that this personalized planning approach could reduce clinically relevant ARIE while generally maintaining target coverage. We assessed its clinical feasibility, dosimetric characteristics, and acute toxicity outcomes in patients with lung cancer undergoing definitive TRT who were considered at high risk for ARIE. Moreover, we further conducted exploratory analyses of potential factors associated with esophagitis.

## Methods and materials

### Patient selection

Patients diagnosed with histologically confirmed lung cancer who underwent definitive TRT with a biologically effective dose (BED) of at least 50 Gy between May 2023 and January 2025 at one institution were enrolled. Eligible patients had a shortest distance between the clinical target volume (CTV) and the esophagus less than 5 mm. Some patients were recruited from the prospective trials iLCC-3 (NCT05557552, registered on 28 September 2022) [24] and iLCC-4 (NCT05552846, registered on 23 September 2022). This study was conducted in accordance with the Declaration of Helsinki and approved by the Institutional Review Board at Shanghai Ruijin Hospital. All patients provided written informed consent.

To offer supplementary context for the observed ARIE outcomes, a pre-ES historical benchmark cohort was retrospectively identified. Consecutive patients treated at our institution between January 2022 and April 2023, who met the same predefined high-risk criterion as used in the present study and received standard-planning TRT, were screened. No patients were selectively excluded. This historical cohort was incorporated to provide an institutional reference for ARIE incidence under generally similar supportive care practices, rather than as a formally matched control group for efficacy comparison.

For the supplementary paired dosimetric comparison, a stratified consecutive sample of 10 patients was selected from the study cohort to represent the major fractionation schedules used in this study. Specifically, the sample included 3 patients treated with 45 Gy in 30 fractions twice daily, 3 patients treated with 45 Gy in 15 fractions, 2 patients treated with 50 Gy in 25 fractions, and 2 patients treated with 60 Gy in 30 fractions. Within each fractionation stratum, the first 2 to 3 consecutive eligible patients were included. For each selected patient, a paired standard plan was generated for dosimetric comparison with the ES-plan.

The patient selection flow is shown in Supplementary Fig. 1.

### Treatment

Definitive TRT incorporating the ES technique was planned for all patients. Treatment was planned with intensity-modulated radiation therapy (IMRT) or volumetric-modulated arc therapy (VMAT) (Eclipse; Varian) using contrast enhanced, custom immobilization, 4-dimensional CT planning with a 3 mm slice thickness, and daily image guidance.

Gross tumor volume (GTV) was delineated based on the tumor observed on PET/CT and/or contrast-enhanced CT. To account for respiratory motion, the internal target volume (ITV) was generated by encompassing the GTV across all respiratory phases. The CTV was created by expanding the ITV isotropically by 5–8 mm, adjusted according to tumor histology and avoiding adjacent OARs. A uniform 5 mm margin was added to the CTV in all directions to define the PTV. All OARs were delineated according to the in-house guidelines.

Treatment plans were accepted if the D95 of the target volume exceeded 99% of the prescribed dose (or showed only clinically acceptable deviation) and all OAR constraints were satisfied. Dose constraints for OARs were: (1) spinal cord maximum dose < 40 Gy; (2) heart mean dose < 20 Gy; (3) ipsilateral lung V30_Gy_ < 30%, V20 _Gy_ < 50%, and V5 _Gy_ < 70%; (4) lungs V30 _Gy_ < 12%, V20 _Gy_ < 20%, and V5 _Gy_ < 45%; (4) lungs mean dose < 15 Gy. Systemic treatment (including chemotherapy, targeted therapy or immunotherapy) was administered according to multidisciplinary team (MDT) recommendations.

### ES technique

**Fig. 1:**
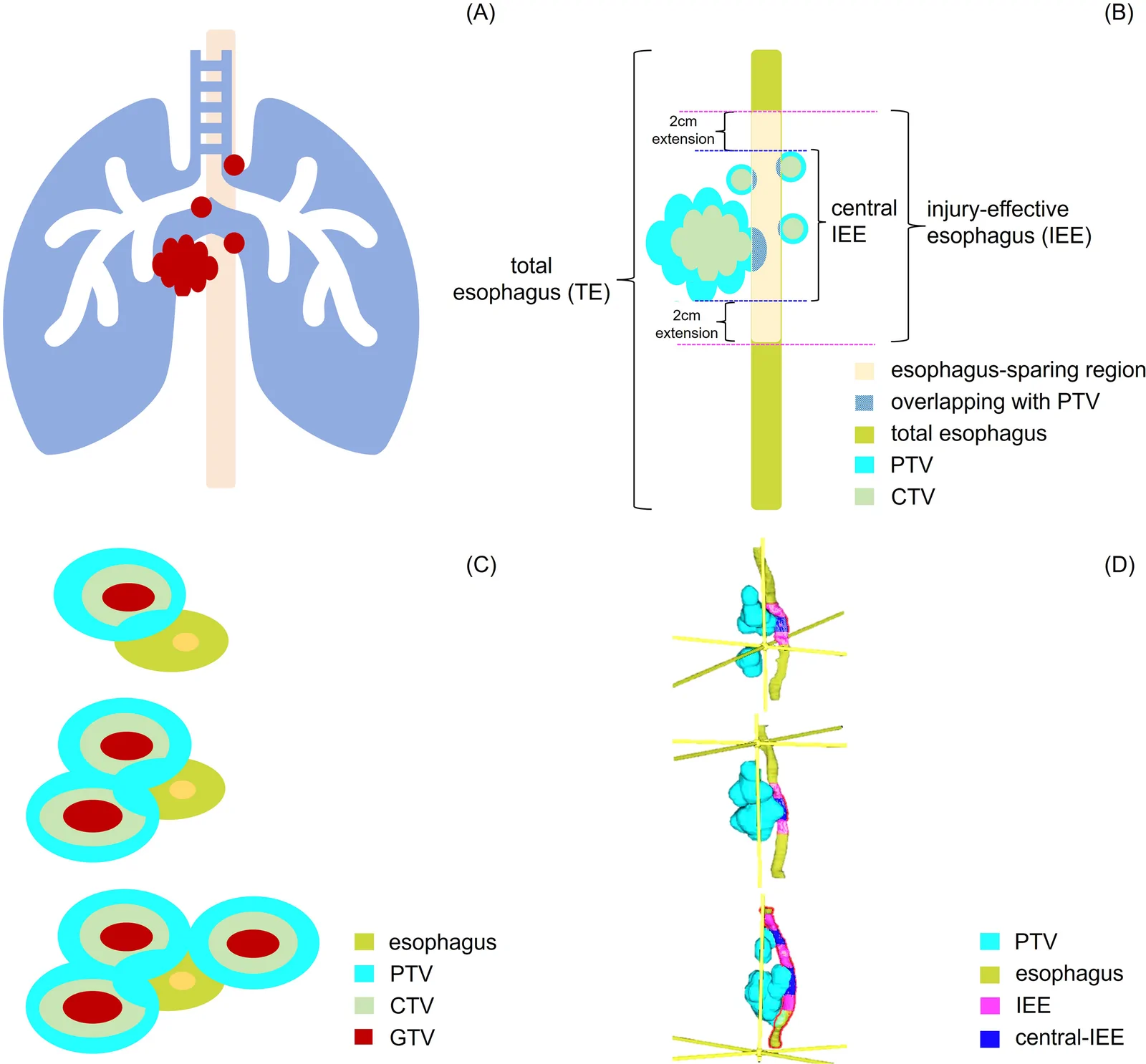
Schematic illustration of the esophagus-sparing (ES) technique. (**A**) Conceptual overview of the local geometric relationship between the tumor and the esophagus in thoracic radiotherapy. (**B**) Definition of the IEE, central IEE and the ES region. (**C**) Abstract axial-view schematic illustrations of the three categories of PTV/central-IEE overlap are shown, from top to bottom, as < 1/3, 1/3–2/3, and > 2/3, respectively. (**D**) Patient-based three-dimensional schematic views of the same three categories are shown from top to bottom as < 1/3, 1/3–2/3, and > 2/3, respectively, and illustrate the spatial relationships among the PTV, esophagus, IEE, and central-IEE. (**C**) and (**D**) are complementary visual representations of the same classification framework. In the actual planning process, category assignment was determined by the volumetric overlap ratio between the PTV and the central IEE, rather than by assessment at a single axial level. These categories were used to assign graded ES-Dmean constraints during treatment planning

A novel segmental contralateral ES technique was developed and implemented during treatment planning. The “contralateral” refers to the esophageal side opposite the target interface within the relevant local segment [21, 22]. The IEE was defined as the local high-risk esophageal segment adjacent to the target, consisting of the central-IEE and an additional 2 cm superior and inferior cranio–caudal expansion. The central-IEE was defined as the contiguous core axial segment of the esophagus encompassing all axial levels where overlap between the PTV and the esophagus was present. The ES region was contoured as the esophageal segment within IEE excluding any direct overlap with the PTV (detailed in Fig. 1).

Target coverage and conventional OAR constraints were prioritized over ES constraints. To balance esophageal protection with target coverage, mean dose to the ES region (ES-Dmean) was determined based on the proportion of the central-IEE volume overlapped by the PTV. Specifically, when the PTV overlapped with < 1/3, 1/3–2/3, or > 2/3 of the central-IEE, the corresponding ES-Dmean constraint was set at 1/3, 1/2, or 2/3 of the prescribed dose, respectively. Accordingly, a plan was classified as passing only if it met the relevant ES-Dmean constraint in addition to the standard target coverage and OAR requirements. No predefined acceptable deviation from the ES-Dmean threshold was allowed. Conversely, a plan was classified as failed if it did not meet the assigned ES-Dmean constraint. Additional planning metrics were defined to evaluate ES-plan quality. D_shortest_ referred to the minimum axial-plane edge-to-edge distance between the CTV and the esophagus. P_length−IEE/TE_ and P_volume−IEE/TE_ represented the proportion of the IEE length and volume, respectively, relative to the total esophagus (TE). P_volume−ES/TE_ was similarly calculated. The aforementioned parameters, along with other dosimetric parameters, were collected to assess the dosimetric characteristics of the ES-plan. The linear–quadratic equivalent dose in 2 Gy fractions (EQD2) model was employed, with α/β = 10 for acute esophageal toxicity, α/β = 3 for the heart and lungs, and α/β = 2 for the spinal cord [3, 25, 26]. The EQD2 formula was used to convert dose-volume metrics across different fractionation regimens into a common scale prior to extraction and statistical analysis.

### Toxicity assessment and follow-up

Acute toxicities were graded using the Radiation Therapy Oncology Group (RTOG) criteria, from the initiation of TRT through 3 months after treatment completion. The time to ARIE and other acute toxicities was calculated from the start of TRT to the first occurrence of toxicities of any grade. All patients who developed ARIE received pharmacologic management during routine follow-up to ensure comprehensive supportive care. Late esophageal toxicities were also evaluated using RTOG late toxicity grading criteria during follow-up. Progression-free survival (PFS) was defined as the interval from the initiation of the TRT to the first documented disease progression or death from any cause, whichever occurred first. Overall survival (OS) was defined as the interval from the initiation of the current systemic treatment to death from any cause. Patients alive or without progression at the last follow-up were censored at that date.

### Statistical analysis

The survival probabilities of ARIE, PFS and OS were estimated using the Kaplan–Meier method, and the median follow-up time was calculated using the reverse Kaplan–Meier method. The Wilcoxon signed-rank test was used for paired comparisons between standard plans and ES-plans. Exploratory univariate Cox proportional hazards regression analyses were performed to identify potential risk factors and dose-volume parameters associated with ARIE. The proportional hazards assumption was assessed for each reported univariable Cox model using Schoenfeld residuals. *P*-values were adjusted for multiple testing using the Benjamini-Hochberg (BH) procedure, and an adjusted *P*-value of < 0.05 was considered statistically significant. Hazard ratio (HR) and 95% confidence intervals (CI) were reported. All statistical analyses were performed using R software (version 4.2.2) and GraphPad Prism (version 9.4.1).

## Results

### Patient characteristics

A total of 80 lung cancer patients were enrolled in this study (detailed in Supplementary Fig. 1). The median age at the start of TRT was 68 years (range, 48–80 years). Thirty-three patients (41.25%) were diagnosed with NSCLC, including 12 who were ineligible for concurrent chemo-radiation therapy and participated in the iLCC-3 trial [24]. The remaining 47 patients (58.75%) had SCLC, including 18 with extensive-stage disease (ES-SCLC) who were enrolled in the iLCC-4 trial. The median BED was 58.5 Gy (range, 51.75–72 Gy), assuming an α/β ratio of 10. Concurrent chemo-radiation therapy was administered to 48 patients (60.0%), while 23 patients (28.8%) received concurrent CIRT. Detailed baseline characteristics are summarized in Table 1.

**Table 1:** Patient and treatment characteristics in this cohort (*n* = 80)

Clinical features	No. of patients (%)
**Age (median [range])**,** years**	68 (48–80)
≥ 60	70 (87.5%)
<60	10 (12.5%)
**Gender**	
Female	14 (17.5%)
Male	66 (82.5%)
**Clinical AJCC stage (8th edition)**	
II	5 (6.26%)
IIIA	15 (18.75%)
IIIB	19 (23.75%)
IIIC	17 (21.25%)
IV	24 (30.0%)
**ECOG performance status**	
0 ~ 1	72 (90.0%)
2	8 (10.0%)
**Smoking history**	
Yes	56 (70.0%)
No	24 (30.0%)
**Comorbidities**	
Yes	51 (63.75%)
No	29 (36.25%)
**Histologic type**	
NSCLC	33 (41.25%)
SCLC	47 (58.75%)
**Tumor laterality**	
Left and mediastinal	40 (50.0%)
Right and mediastinal	32 (40.0%)
Mediastinal	8 (10.0%)
**Chemotherapy**	
Concurrent chemotherapy	49 (61.25%)
Sequential chemotherapy	28 (35.0%)
No chemotherapy	3 (3.75%)
**Concurrent immunotherapy**	
Yes	47 (58.75%)
No	33 (41.25%)
**Prescribed dose**	
45 Gy in 15 fractions, QD	24 (30.0%)
45 Gy in 30 fractions, BID	24 (30.0%)
50 Gy in 2 Gy/fraction, QD	15 (18.75%)
56 Gy in 2 Gy/fraction, QD	1 (1.25%)
60 Gy in 2 Gy/fraction, QD	16 (20.0%)
**BED (median [range])**,** Gy**	58.5 (51.8–72.0)
50 Gy ≤ BED < 60 Gy	48 (60.0%)
60 Gy ≤ BED < 70 Gy	16 (20.0%)
BED ≥ 70 Gy	16 (20.0%)
**Technology type**,** n (%)**	
IMRT	72 (90.0%)
VMAT	8 (10.0%)

Abbreviation: AJCC, American Joint Committee on Cancer; ECOG, Eastern Cooperative Oncology Group; NSCLC, non-small cell lung cancer; SCLC, small cell lung cancer; QD, quaque die, once a day; BID, bis in die, twice a day; BED, biologically effective dose

### Dosimetric parameters of the segmental contralateral ES technique

**Fig. 2:**
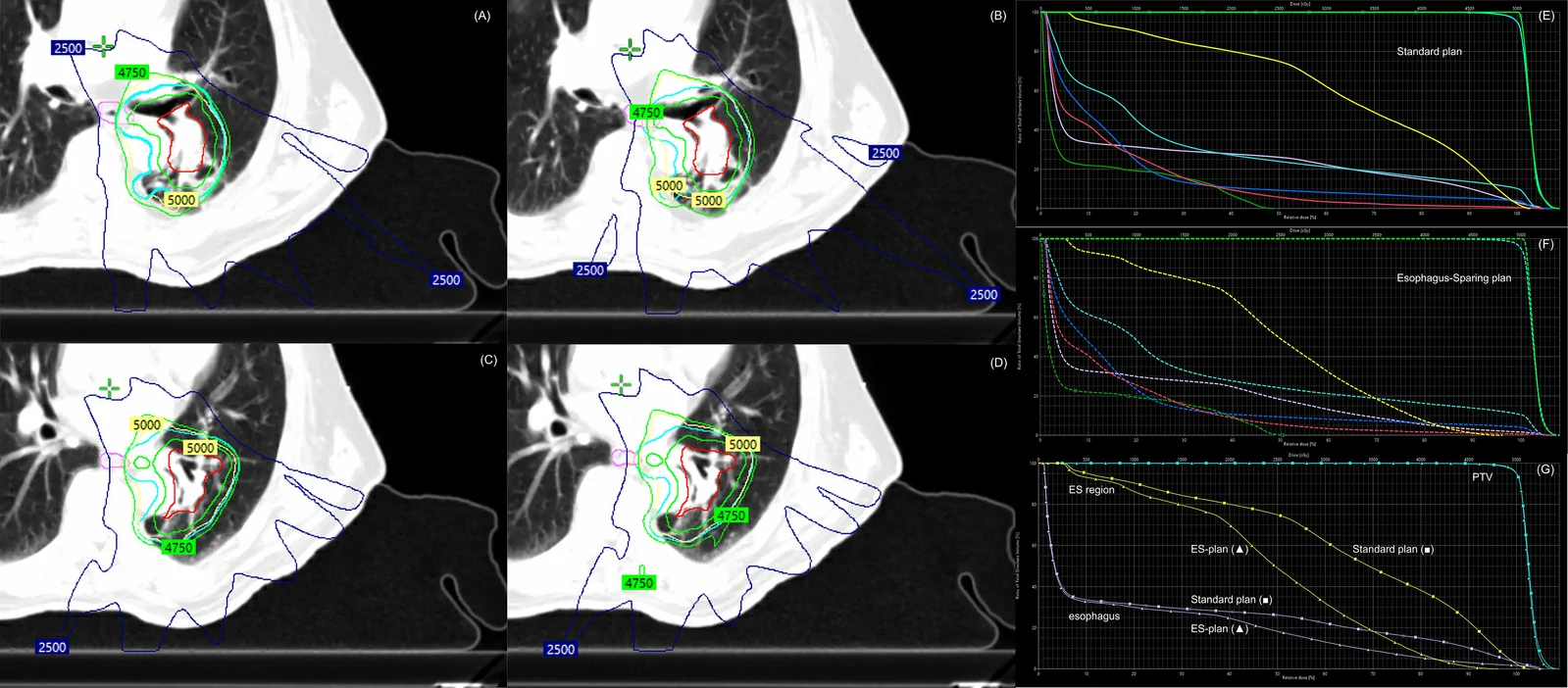
Illustrating the ES technique applied in a patient with NSCLC (axial CT Images) (**A**) and (**C**) Examples of a representative patient undergoing standard treatment planning. PTV (cyan), CTV (green), GTV (red), esophagus (pink), esophagus-sparing region (yellow). (**B**) and (**D**) Examples of the same representative patient undergoing ES-strategy planning. PTV (cyan), CTV (green), GTV (red), esophagus (pink), esophagus-sparing region (yellow). (**E**) Dose-volume histogram of this patient undergoing standard treatment planning. PTV (cyan), CTV (green), esophagus (pink), esophagus-sparing region (yellow), right lung (light cyan), lungs (blue), heart (red), spinal cord (dark green). (**F**) Dose-volume histogram of this patient undergoing ES-strategy planning. PTV (cyan), CTV (green), esophagus (pink), esophagus-sparing region (yellow), right lung (light cyan), lungs (blue), heart (red), spinal cord (dark green). (**G**) Dose-volume histogram comparison of the same patient undergoing standard treatment planning (■) and ES-strategy planning (▲), showing PTV (cyan), esophagus-sparing region (yellow) and esophagus (pink)

All 80 enrolled patients had a D_shortest_ less than 5 mm and were eligible for ES radiation therapy planning. The ES-plans were successfully generated for all cases. A total of 77 patients (96.25%) achieved coverage of 95% of the PTV with more than 99% of the prescribed dose, while the remaining 3 patients achieved D95 with more than 98% of the prescribed dose. Target coverage and conventional OAR dose constraints were maintained in all cases. Dosimetric data are summarized in Table 2. The dose distribution for a representative patient before and after application of the ES technique is illustrated in Fig. 2. However, considering the clinical priority settings, eleven patients (13.75%) did not fully meet the requirements of the ES radiation therapy plan in the final treatment plans (actual dosimetric parameters shown in Supplementary Table 1).

**Table 2:** Dosimetric parameters of the esophagus-sparing radiotherapy plans

Details of the Esophagus-Sparing RT plans	Value
**Part I: dosimetric parameters of the target volume & conventional OAR**	
**CTV (median [range])**,** cm**^**3**^	96.45 (1.70–376.47)
**PTV (median [range])**,** cm**^**3**^	214.93 (17.20–655.81)
**PTV D95 > 99%PD**	96.25%
**Ipsilateral lung dosimetric parameters**	
Dmean (mean/median [IQR]), cGy	1622.7 / 1574.7 (1338.1–1879.0)
V5_Gy_ (mean [IQR]), %	59.12 (52.09–66.80)
V20_Gy_ (mean [IQR]), %	33.18 (27.26–39.51)
**Lungs Dmean (mean/median [IQR])**,** cGy**	1076.3 / 1034.1 (873.6–1275.5)
**Heart dosimetric parameters**	
Dmean (mean/median [IQR]), cGy	811.3 / 704.7 (367.0–1237.4)
V40_Gy_ (mean [IQR]), %	4.88 (0.53–7.14)
**Spinal cord Dmax (mean/median [IQR])**,** cGy**	3242.0 / 3417.1 (2937.4–3811.3)
**Part II: general parameters of the ES-plan**	
**Prescribed ES type**,** n (%)**	
ES-Dmean ≤ 1/3 PD	28 (35.0%)
ES-Dmean ≤ 1/2 PD	18 (22.5%)
ES-Dmean ≤ 2/3 PD	34 (42.5%)
**Pass rate of each ES type**,** n (%)**	Total pass rate: 86.25%
ES-Dmean ≤ 1/3 PD	64.3% (18/28)
ES-Dmean ≤ 1/2 PD	94.4% (17/18)
ES-Dmean ≤ 2/3 PD	100.0% (34/34)
**D**_**shortest**_ **(median [IQR])**,** mm**	0 (0–0)
**P**_**Length−IEE/TE**_ **(mean [IQR])**,** %**	56.73 (47.50–63.75)
**P**_**Volume−ES/TE**_ **(mean [IQR])**,** %**	46.52 (36.92–53.51)
**P**_**Volume−IEE/TE**_ **(mean [IQR])**,** %**	53.63 (45.19–60.65)
**Part III: dosimetric parameters of the ES-plan**	
**Esophagus-Sparing area dosimetric parameters**	
Dmax (mean/median [IQR]), cGy	5079.7 / 5060.7 (4571.2–5272.0)
Dmean (mean/median [IQR]), cGy	2250.0 / 2155.5 (1657.3–2865.8)
V20_Gy_ (mean [IQR]), %	49.42 (33.06–65.86)
V30_Gy_ (mean [IQR]), %	34.61 (16.37–50.61)
V40_Gy_ (mean [IQR]), %	19.26 (5.33–31.98)
V50_Gy_ (mean [IQR]), %	4.13 (0–3.38)
V60_Gy_ (mean [IQR]), %	0.71 (0–0)
**Total Esophagus dosimetric parameters**	
Dmax (mean/median [IQR]), cGy	5322.0 / 5269.5 (4794.7–5430.2)
Dmean (mean/median [IQR]), cGy	1582.6 / 1471.8 (1147.5–1942.3)
V20_Gy_ (mean [IQR]), %	31.57 (22.30–41.05)
V30_Gy_ (mean [IQR]), %	24.69 (14.55–33.23)
V40_Gy_ (mean [IQR]), %	17.49 (8.01–25.18)
V50_Gy_ (mean [IQR]), %	6.82 (0–9.65)
V60_Gy_ (mean [IQR]), %	2.04 (0–0)

Abbreviation: RT, radiation therapy; IMRT, intensity-modulated radiation therapy; VMAT, volumetric modulated arc therapy; CTV, clinical target volume; PTV, planning target volume; PD, prescribed dose; ES, esophagus-sparing; D_shortest_, the shortest distance between CTV and esophagus in the axial section; P_Length_, the percentage of length; P_Volume_, the percentage of volume; IEE, injury-effective esophagus; TE, total esophagus

### ARIE and other acute toxicities

All patients completed the full course of TRT and were followed up for a minimum of 3 months after treatment completion. ARIE was observed in 24 patients (30.0%). The median time to ARIE onset was 21 days (range, 14–74 days). Among these, fifteen cases were classified as Grade 1 ARIE, eight cases as Grade 2 ARIE, and one case as Grade 3 ARIE. No instances of Grade 4 or higher ARIE were reported. Additionally, no late esophageal toxicities were observed during the follow-up time. Exploratory analyses of the incidence of ARIE across major clinical and treatment subgroups are presented in Supplementary Table 2. Given the limited sample size, these analyses were intended to evaluate the consistency of the observed ARIE pattern rather than to support formal subgroup inferences. A detailed summary of treatment-related acute toxicities is presented in Table 3.

**Table 3:** Acute radiation-induced esophagitis and other acute toxic events

Toxic events (RTOG)	Grade 1, *n* (%)	Grade 2, *n* (%)	Grade 3, *n* (%)	Grade 4, *n* (%)	Grade 5, *n* (%)	Total events, *n* (%)
Esophagitis	15 (18.75%)	8 (10.0%)	1 (1.25%)	0 (0%)	No Grade 5 toxic events happened.	24 (30.0%)
Pneumonitis ^a^	16 (20.0%)	13 (16.25%)	2 (2.5%)	0 (0%)		31 (38.75%)
Upper-GI injury ^b^	16 (20.0%)	4 (5.0%)	2 (2.5%)	0 (0%)		22 (27.5%)
Hematologic toxicity ^c^	22 (27.5%)	31 (38.75%)	7 (8.75%)	1 (1.25%)		61 (76.25%)

Abbreviation: RTOG, Radiation Therapy Oncology Group; Upper-GI, upper gastrointestinal

^a^ pneumonitis caused by radiotherapy and/or concurrent immunotherapy; ^b^ upper-GI injury caused by radiotherapy and/or concurrent chemotherapy and/or concurrent immunotherapy; ^c^ hematologic toxicity caused by radiotherapy and/or concurrent chemotherapy and/or concurrent immunotherapy, including leukopenia, neutropenia, thrombocytopenia, and anemia

Among the 80 patients in the full cohort, eleven patients did not fully meet the predefined ES dose constraints. A per-protocol analysis was therefore performed in the remaining 69 patients. In this cohort, the incidence of any-grade ARIE, Grade 1–2 ARIE, and severe ARIE (Grade ≥ 3) was 26.1%, 24.6%, and 1.4%, respectively, which was similar to that observed in the full cohort. The overall pattern of other acute toxicities was also generally comparable (Supplementary Table 3).

### Historical benchmark and paired dosimetric comparison

To better contextualize the observed ARIE incidence, a pre-ES historical cohort was included as a benchmark. A total of 51 cases were included in the historical cohort (Supplementary Table 4). For contextual reference, the incidence of any-grade ARIE, Grade 1–2 ARIE, and severe ARIE (Grade ≥ 3) in the pre-ES cohort was 76.5%, 68.6%, and 7.8%, respectively. As this historical cohort was neither formally matched nor included in the study for any purpose other than background analysis, these comparisons should be interpreted with caution.

Second, paired replanning was carried out in a stratified consecutive sample of 10 patients from the study cohort to compare standard planning with ES-planning. Compared with standard planning, ES-planning largely preserved basic target coverage. There were no significant differences in PTV D95(percentage of prescribed dose), D90 (percentage of prescribed dose), or D2 (percentage of prescribed dose) between the standard and ES-plans, although V95% exhibited a marginal decrease. Nevertheless, ES-plans showed significantly poorer homogeneity, reflected by a higher homogeneity index. Regarding OARs, ES-planning significantly reduced the mean dose to the total esophagus (2138.03 cGy vs. 1833.80 cGy, *p* = 0.0371) and mean dose to the ES region (3218.96 cGy vs. 2451.16 cGy, *p* = 0.0039), along with several low-to-intermediate dose-volume metrics. No statistically significant increase in heart or spinal cord dose was observed, and ipsilateral lung dose metrics remained broadly comparable between the two plans. Detailed dosimetric results are presented in Table 4.

**Table 4:** Dosimetric comparison between the standard plans and the ES-plans

PTV & OAR	Dose-Volume metrics	Standard plan	ES-plan	*P*-value
PTV	D95% (% of PD), median (IQR)	100.00% (100%- 100%)	100.00% (99.11% – 100.00%)	0.1250
	D90% (% of PD), median (IQR)	100.73% (100.48% – 100.98%)	100.54% (100.00% – 100.88%)	0.7422
	D2% (% of PD), median (IQR)	106.02% (105.20% – 106.60%)	106.84% (105.89% – 107.96%)	0.0645
	V107%, median (IQR)	0.40% (0.10% – 1.25%)	0.65% (0.42% – 1.28%)	0.8457
	V95%, median (IQR)	99.75% (99.70% – 99.90%)	99.65% (99.45% – 99.85%)	0.0547
	conformity index	1.19 (1.14–1.21)	1.07 (1.06–1.09)	0.0195*
	homogeneity index, median (IQR)	0.07 (0.06–0.08)	0.09 (0.08–0.10)	0.0098*
Ipsilateral Lung	Dmean (cGy), median (IQR)	1482.06 (1214.48–1838.18)	1499.58 (1252.22–1851.17)	0.7695
	V5 _Gy_, median (IQR)	55.60% (47.18% – 67.53%)	58.21% (45.86% – 67.72%)	0.4473
	V20 _Gy_, median (IQR)	29.00% (25.88% – 39.60%)	28.03% (24.47% – 37.99%)	0.4316
Lungs	Dmean (cGy), median (IQR)	893.55 (810.35–1177.62)	1075.00 (855.21–1374.60)	0.0645
Heart	Dmean (cGy), median (IQR)	502.82 (391.17–1180.76)	530.70 (350.48–1157.40)	0.7695
	V40 _Gy_, median (IQR)	2.50% (0.75% – 5.85%)	3.12% (0.73% – 5.33%)	0.8203
Spinal Cord	Dmax (cGy), median (IQR)	3464.70 (3068.20–4035.64)	3519.55 (3234.19–4059.28)	0.0840
Total Esophagus	Dmax (cGy), median (IQR)	5242.79 (4805.34–5400.84)	5269.49 (4974.80–5375.86)	0.6250
	Dmean (cGy), median (IQR)	2138.03 (1633.53–2271.84)	1833.80 (1393.39–2126.69)	0.0371*
	V20 _Gy_, median (IQR)	42.85% (38.50% – 45.28%)	39.05% (28.26% – 43.75%)	0.3750
	V30 _Gy_, median (IQR)	37.45% (23.58% – 41.70%)	33.59% (18.04% – 38.80%)	0.0645
	V40 _Gy_, median (IQR)	29.35% (13.93% – 36.48%)	24.73% (10.45% – 35.06%)	0.0059*
	V50 _Gy_, median (IQR)	9.25% (0.60% – 21.30%)	3.86% (0.77% – 6.70%)	0.0781
	V60 _Gy_, median (IQR)	0% (0–0)	0% (0–0)	0.5000
Esophagus sparing area	Dmax (cGy), median (IQR)	5206.34 (4766.78–5371.41)	5121.18 (4734.29–5283.26)	0.0645
	Dmean (cGy), median (IQR)	3218.96 (2747.05–3648.06)	2451.16 (2124.85–3361.30)	0.0039*
	V20 _Gy_, median (IQR)	74.64% (61.65% – 82.10%)	59.33% (44.47% – 75.79%)	0.0098*
	V30 _Gy_, median (IQR)	59.49% (49.80% – 71.08%)	42.82% (36.42% – 62.79%)	0.0098*
	V40 _Gy_, median (IQR)	38.15% (20.60% – 56.05%)	25.47% (10.91% – 44.60%)	0.0039*
	V50 _Gy_, median (IQR)	5.85% (0.33% – 26.20%)	0.12% (0–1.68%)	0.0156*
	V60 _Gy_, median (IQR)	0% (0–0)	0% (0–0)	> 0.9999

Abbreviations: PTV, planning target volume; OAR, organ at risk; ES, esophagus-sparing; PD, prescribed dose

### Exploratory assessment of potential factors related to ARIE

Exploratory univariable analyses were performed to explore potential clinical, geometric, treatment-related, and dosimetric factors associated with ARIE. In addition to the predefined high-risk geometric criterion of close CTV-esophagus proximity, exploratory associations with ARIE were observed for concurrent CIRT (HR = 2.46 [95%CI, 1.10–5.51], *p* = 0.028), P_volume−ES/TE_ (HR = 1.28 per 5% increase [95%CI, 1.08–1.52], *p* = 0.004), passing the ES-plan (HR = 0.37 [95%CI 0.15–0.93], *p* = 0.035), 3 Gy per fraction (HR = 3.09 [95%CI, 1.38–6.88], adjust-*p* = 0.018), and total mean dose of the esophagus (HR = 1.06 per 1 Gy increase [95%CI, 1.01–1.12], *p* = 0.037) (Supplementary Table 5). Given the limited sample size and event number, these findings are hypothesis-generating only and should be interpreted cautiously.

### Descriptive oncologic outcomes

At a median follow-up time of 22.0 months (95%CI, 18.7–25.1), the 1- and 2-year OS rates for the entire cohort were 88.8% (95% CI, 82.1–96.0) and 68.9% (95% CI, 58.4–81.4), respectively (Supplementary Fig. 2). Among the 25 deaths observed, eleven occurred in patients with ES-SCLC, and two had received third-line treatment prior to definitive TRT.

Median PFS, defined from initiation of radiotherapy, was 11.7 months (95% CI, 8.6 – NA) (Supplementary Fig. 3). Recurrence pattern analysis identified 7 patients with in-field or marginal recurrence, including 3 with concurrent out-of-field intrapulmonary lesions and 2 with synchronous distant metastases. Subgroup survival curves are provided in Supplementary Figs. 2 and 3 for descriptive completeness only and should be interpreted cautiously given the heterogeneous treatment contexts in this cohort.

## Discussion

ARIE remains one of the most common toxicities in lung cancer patients receiving TRT and can substantially impair the quality of life. As a dose-limiting toxicity, escalation of the prescribed TRT dose may result in greater radiation exposure to the esophagus, thereby increasing the risk of ARIE [8, 27]. The landmark study RTOG 0617 identified the maximum grade of esophagitis as an independent prognostic factor for overall survival [1], underscoring the importance of effective ES strategies in modern TRT planning. In this study, we developed a novel segmental contralateral ES strategy that appeared technically feasible and achieved region-focused esophageal dose reduction. In this single-arm cohort, the ES-strategy was associated with a favorable acute esophageal toxicity profile, with 30.0% of patients experiencing any-grade of ARIE and only one Grade 3 event. The basic target coverage was generally maintained, although some trade-offs between dose homogeneity and OAR constraints were observed in the paired dosimetric comparison. The observed ARIE rate also appears broadly consistent with prior studies that applied different ES strategies [8, 13, 15, 21, 28], although such cross-study comparisons should be interpreted cautiously.

Previous studies have investigated various ES strategies in the context of TRT (summarized in Supplementary Table 6). Some approaches concentrated on reducing radiation dose to the contralateral esophagus [21–23], while others aimed to achieve a steep dose falloff outside the PTV to minimize esophageal exposure [15–20]. Many of these studies were conducted in NSCLC patients receiving dCCRT, and generally required narrow target–esophagus margins (typically 5–10 mm). Although these approaches, typically implemented through advanced IMRT techniques, were associated with favorable reductions in the incidence of ARIE, several limitations remain. In contrast, our segmental contralateral ES-technique differs in several key aspects. First, it is a region-specific sparing strategy that focuses on the local high-risk esophageal segment adjacent to the PTV. Second, it incorporates individualized ES dose constraints based on the degree of PTV/central-IEE overlap, offering a more flexible and risk-adapted planning framework. Third, instead of relying on a single fixed dose-volume metric, this dose-optimized strategy can be applied across different prescription schedules and might be more practical for routine clinical planning. Collectively, these features indicate that the current strategy expands upon prior ES concepts to create a more clinically adaptable planning workflow.

A critical aspect of implementing an ES strategy in TRT is precisely defining both the locally relevant esophageal segment and a clinically meaningful dose constraint. In this study, we introduced the concepts of the IEE and central-IEE, and delineated the ES region as the portion of the IEE outside the PTV. The mean dose constraint for the ES region was then individualized according to the degree of PTV/central-IEE overlap, as well as the patient-specific prescription dose. The rationale for introducing the IEE concept is that previous studies suggest that the esophageal region most relevant to ARIE is likely to be localized rather than encompassing the entire esophagus. This is supported by evidence of regional dose heterogeneity along the superior–inferior axis [29, 30], the importance of small high-dose esophageal subvolumes [8, 31, 32], and the viability of segmental ES approaches [15]. The mean dose was selected as the principal ES constraint because it has been consistently associated with the risk of ARIE in previous studies [8, 13, 33–35], comprehensively reflects the cumulative dose burden across the spared esophageal region compared to a single point-based metric, and is less sensitive to inter-fractional and intra-fractional esophageal motion. Collectively, these considerations support the use of the mean ES dose as a practical and clinically meaningful planning metric for the ES strategy employed in this study.

Another significant challenge in interpreting the present study is the lack of a formal control group, which we recognize as a major limitation. To partially address this issue, we reviewed a pre-ES historical cohort of patients who met the same predefined high-risk criterion for ARIE. Since institutional supportive care practices for ARIE remained essentially unchanged during this period, this cohort was used as a historical benchmark for ARIE incidence. The observed incidence of ARIE in the ES cohort seemed to be more favorable compared to that in this benchmark cohort. In addition, to provide quantitative support for the dosimetric effect of the ES strategy beyond a single representative case, we conducted a paired comparison between the standard plan and the ES-plan in 10 patients with varying prescription schedules. This analysis demonstrated that the ES strategy decreased the mean dose to both the ES region and the total esophagus while generally maintaining target coverage. Nevertheless, the historical cohort did not serve as a formally matched comparative control, and the dosimetric study was carried out on a representative subset instead of the entire cohort. Accordingly, these supplementary analyses should be regarded as supportive rather than conclusive; prospective comparative validation will still be required to more rigorously establish the clinical advantages of the ES strategy.

Several limitations should be acknowledged. Beyond the lack of a formal control group, as discussed above, this was a single-center, retrospective study involving a relatively small and clinically heterogeneous cohort. Although this heterogeneity partly reflected our intention to evaluate the ES strategy across a broader spectrum of definitive TRT settings, it also diminished the interpretability and generalizability of the toxicity findings. Second, the use of an α/β ratio of 10 Gy for ARIE was based on conventional assumptions rather than esophageal-specific radiobiological data, especially when a portion of the esophagus is included within the PTV. This may have influenced dose conversion across fractionation schemes. Finally, the current follow-up is still insufficient for a robust assessment of late esophageal toxicity, and patient-reported outcomes were unavailable, which may have limited symptom-level assessment. Even so, this study achieved its primary objective of validating the feasibility of the ES strategy and presenting preliminary dosimetric data and evidence of acute toxicity.

Despite these limitations, the present study supports the technical feasibility of a practical, individualized esophagus-sparing strategy for patients with lung cancer who are undergoing definitive TRT with esophagus–PTV abutment. By incorporating risk-adapted dose constraints based on the local spatial relationships between the PTV and the esophagus, this strategy achieved region-focused esophageal dose reduction and was associated with a favorable acute esophageal toxicity profile while broadly maintaining target coverage. These findings should primarily be interpreted within the framework of feasibility, dosimetric effects, and acute toxicity. Prospective comparative validation with longer follow-up will be necessary to more comprehensively define the long-term clinical value of this strategy.

## Supplementary Information

Below is the link to the electronic supplementary material.

Supplementary Material 1

Supplementary Material 2

## Data Availability

Research data are stored in an institutional repository and will be available from the corresponding author upon reasonable request.
