# Supplementary material for: A risk-adapted, dose-optimized esophagus-sparing radiation therapy technique for high-risk patients with lung cancer: feasibility, dosimetry, and acute esophageal toxicity
Source: Radiat Oncol. 2026 Jun 23;21:123. doi: 10.1186/s13014-026-02880-3 (PMC13548649; doi:10.1186/s13014-026-02880-3)

Supplementary Figures

Supplementary Figure 1. The study scheme for the patient selection flow. (page2)

Supplementary Figure 2. Overall survival of the study cohort. (page3)

Supplementary Figure 3. Progression-free survival of the study cohort. (page4)

Supplementary Figure. 1. The study scheme for the patient selection flow.

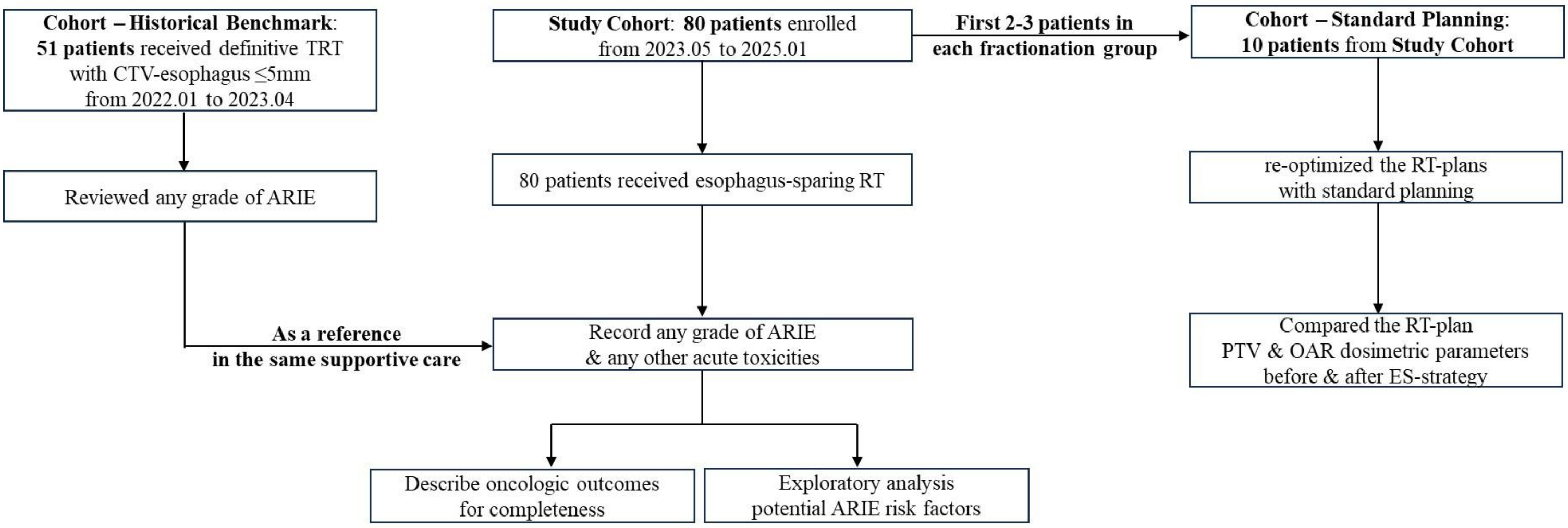

Supplementary Figure 2. Overall survival of the study cohort.

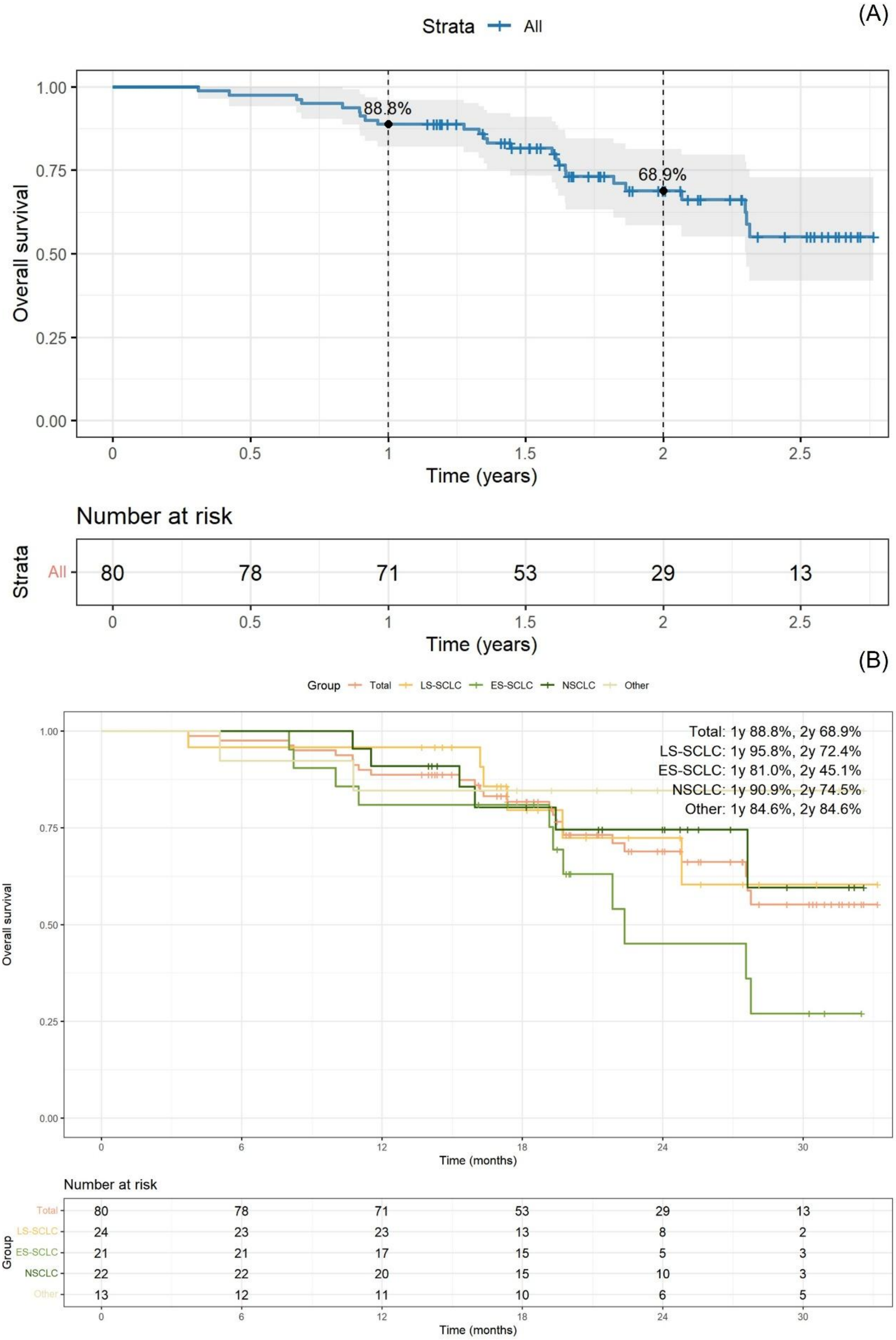

Supplementary Figure 3. Progression-free survival of the study cohort.

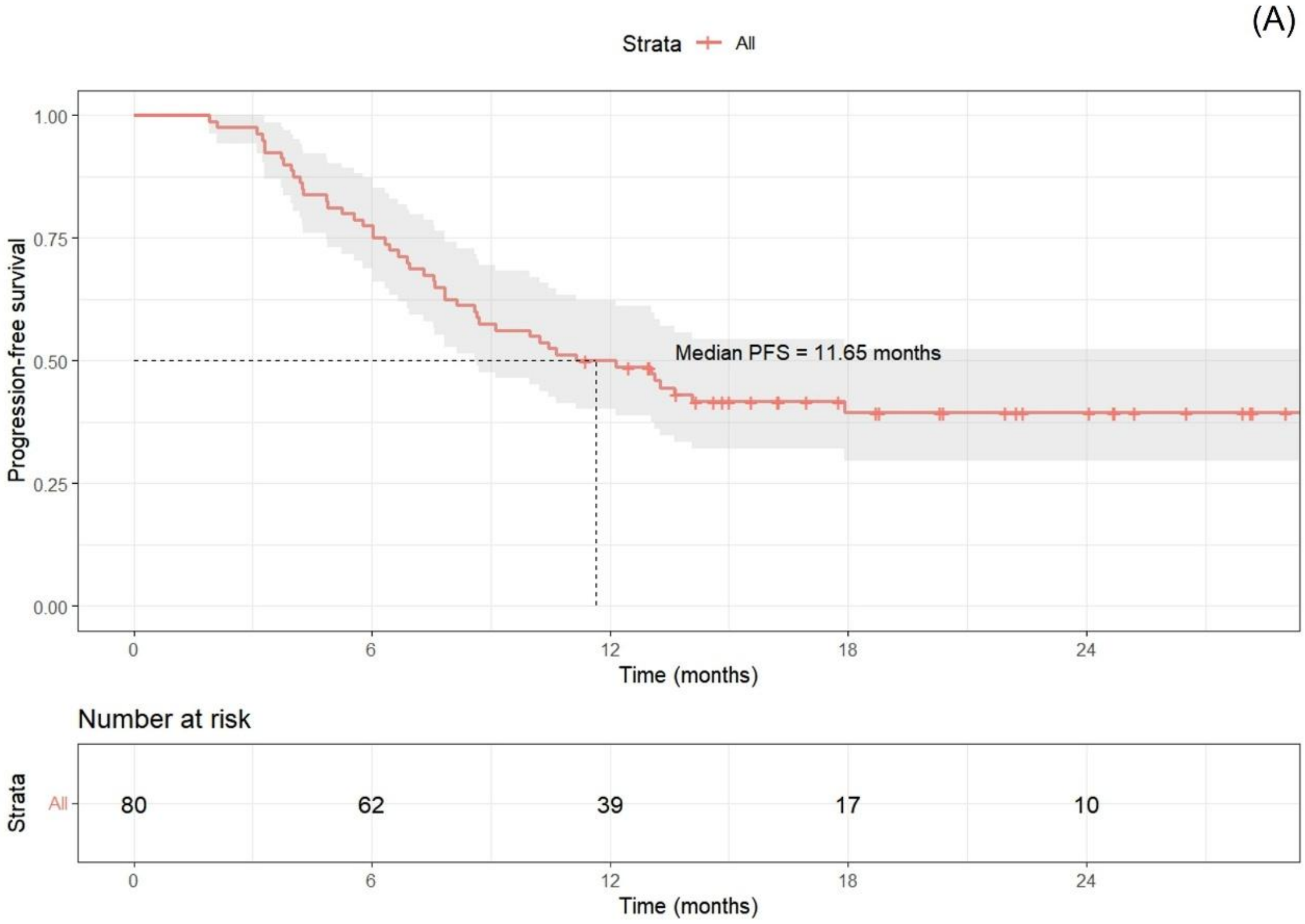

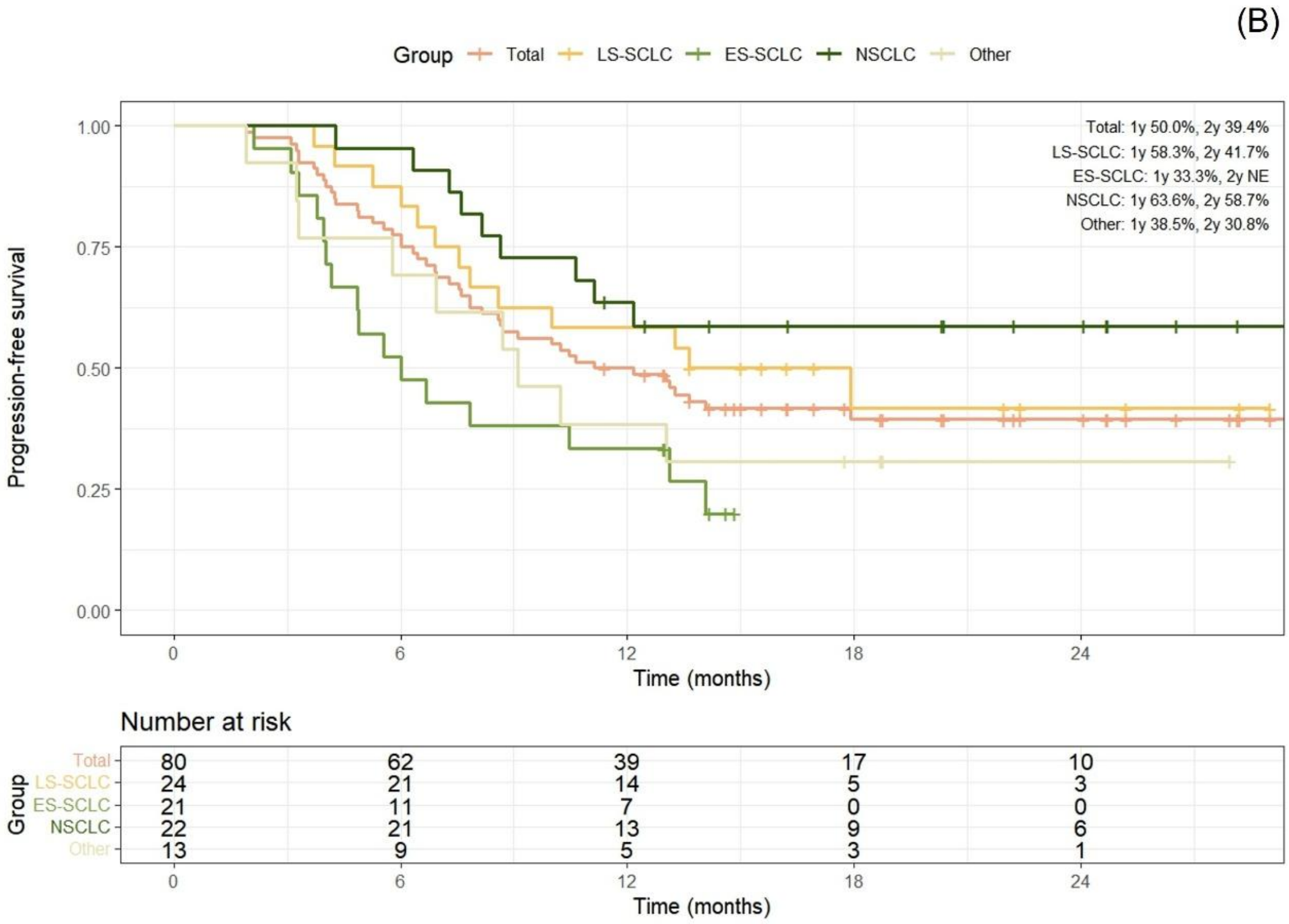

Supplement: Supplementary file 1 [file 13014_2026_2880_MOESM1_ESM.pdf]
