# Supplementary material for: A risk-adapted, dose-optimized esophagus-sparing radiation therapy technique for high-risk patients with lung cancer: feasibility, dosimetry, and acute esophageal toxicity
Source: Radiat Oncol. 2026 Jun 23;21:123. doi: 10.1186/s13014-026-02880-3 (PMC13548649; doi:10.1186/s13014-026-02880-3)
Supplement: Supplementary file 2 [file 13014_2026_2880_MOESM2_ESM.pdf]

Supplementary Tables

Supplementary Table 1. Dosimetric parameters of the actual plans that did not meet the ES-constraints. (page 2)

Supplementary Table 2. Exploratory analyses of ARIE incidence across major clinical and treatment subgroups. (page 3)

Supplementary Table 3. Acute toxicities in the per-protocol population (n=69). (page 4)

Supplementary Table 4. Characteristics of the historical cohort (n=51) and the treatment cohort (n=80). (page 5)

Supplementary Table 5. Exploratory assessment of potential factors related to ARIE. (page 6)

Supplementary Table 6. Comparison of studies about Esophagus-sparing techniques. (page 7)

Supplementary Table 1. Dosimetric parameters of the actual plans that did not meet the ES-constraints.

Details of the Esophagus-Sparing RT plans	Value
**Part I: dosimetric parameters of the target volume & conventional OAR**	
**CTV (median [range]), cm^3**	165.30 (59.89 – 318.00)
**PTV (median [range]), cm^3**	346.90 (154.51 – 600.10)
**PTV D95 > 99%PD**	81.82%
**Ipsilateral lung dosimetric parameters**	
Dmean (mean/median [IQR]), cGy	1857.9 / 1792.2 (1431.6 – 2001.1)
$V5_{Gy}$ (mean [IQR]), %	67.29 (59.40 – 75.90)
$V20_{Gy}$ (mean [IQR]), %	38.21 (30.31 – 41.21)
**Lungs Dmean (mean/median [IQR]), cGy**	1322.5 / 1353.2 (1154.7 – 1485.6)
**Heart dosimetric parameters**	
Dmean (mean/median [IQR]), cGy	1204.1 / 1198.6 (777.9 – 1594.2)
$V40_{Gy}$ (mean [IQR]), %	9.34 (3.90 – 15.40)
**Spinal cord Dmax (mean/median [IQR]), cGy**	3518.8 / 3541.9 (3218.9 – 3789.1)
**Part II: general parameters of the ES-plan**	
**Prescribed ES type, n (%)**	
ES-Dmean ≤ 1/3 PD	10 (90.9%)
ES-Dmean ≤ 1/2 PD	1 (9.1%)
ES-Dmean ≤ 2/3 PD	0 (0%)
**Actual ES type, n (%)**	
ES-Dmean ≤ 1/3 PD	0 (0%)
ES-Dmean ≤ 1/2 PD	9 (81.8%)
ES-Dmean ≤ 2/3 PD	2 (18.2%)
**$D_{shortest}$ (median [IQR]), mm**	0 (0 – 0)
**$P_{Length-IEE/TE}$ (mean [IQR]), %**	62.86 (53.12 – 76.10)
**$P_{Volume-ES/TE}$ (mean [IQR]), %**	51.20 (43.10 – 60.74)
**$P_{Volume-IEE/TE}$ (mean [IQR]), %**	62.91 (57.41 – 70.36)
**Part III: dosimetric parameters of the ES-plan**	
**Esophagus-Sparing area dosimetric parameters**	
Dmax (mean/median [IQR]), cGy	5054.6 / 5024.3 (4572.4 – 5310.2)
Dmean (mean/median [IQR]), cGy	2419.4 / 2136.9 (1916.0 – 2966.4)
$V20_{Gy}$ (mean [IQR]), %	52.19 (38.71 – 66.19)
$V30_{Gy}$ (mean [IQR]), %	37.66 (27.67 – 54.57)
$V40_{Gy}$ (mean [IQR]), %	21.33 (11.46 – 30.90)
$V50_{Gy}$ (mean [IQR]), %	5.04 (0 – 3.62)
$V60_{Gy}$ (mean [IQR]), %	0.24 (0 – 0)
**Total Esophagus dosimetric parameters**	
Dmax (mean/median [IQR]), cGy	5297.8 / 5211.1 (4807.3 – 5376.7)
Dmean (mean/median [IQR]), cGy	1982.3 / 1818.4 (1573.8 – 2229.3)
$V20_{Gy}$ (mean [IQR]), %	40.14 (30.96 – 46.40)
$V30_{Gy}$ (mean [IQR]), %	32.93 (22.91 – 40.00)
$V40_{Gy}$ (mean [IQR]), %	24.59 (14.69 – 32.55)
$V50_{Gy}$ (mean [IQR]), %	8.21 (0 – 10.12)
$V60_{Gy}$ (mean [IQR]), %	1.09 (0 - 0)

Abbreviation: RT, radiation therapy; IMRT, intensity-modulated radiation therapy; VMAT, volumetric modulated arc therapy; CTV, clinical target volume; PTV, planning target volume; PD, prescribed dose; ES, esophagus-sparing; $D_{shortest}$, the shortest distance between CTV and esophagus in the axial section; P_{Length}, the percentage of length; P_{Volume}, the percentage of volume; IEE, injury-effective esophagus; TE, total esophagus.

Supplementary Table 2. Exploratory analyses of ARIE incidence across major clinical and treatment subgroups.

Subgroups	ARIE incidence
**Part I: baseline subgroups**	
**Age**	p = 1.00
≥ 60	30.0% (21/70)
<60	30.0% (3/10)
**Gender**	p = 0.749
Female	28.8% (19/66)
Male	35.7% (5/14)
**ECOG performance status**	p = 1.00
0~1	30.6% (22/72)
2	25.0% (2/8)
**Smoking history**	p = 1.00
Yes	30.4% (17/56)
No	29.2% (7/24)
**Technology type, n (%)**	p = 0.097
IMRT	33.3% (24/72)
VMAT	0.0% (0/8)
**Part II: clinical subgroups**	
**Histologic type**	p = 0.805
NSCLC	27.3% (9/33)
SCLC	31.9% (15/47)
**Stage subgroup within SCLC (n = 47)**	p = 0.211
Limited stage SCLC	23.1% (6/26)
Extensive-stage SCLC	42.9% (9/21)
**Part III: treatment-context comparisons**	
**Concurrent chemotherapy**	p = 0.620
Yes	32.7% (16/49)
No	25.8% (8/31)
**Concurrent immunotherapy**	p = 0.082
Yes	38.3% (18/47)
No	18.2% (6/33)
**Concurrent chemo-immuno-radiotherapy**	p = 0.034
Yes	47.8% (11/23)
No	22.8% (13/57)
**Treatment comparison**	p = 0.297
concurrent CRT	32.7% (16/49)
concurrent CIRT	47.8% (11/23)

Data are presented as number of ARIE events/number of patients and incidence (%). P values were calculated using Fisher's exact test and are provided for exploratory descriptive comparison only. No adjustment was made for multiple comparisons. Given the limited sample size and the heterogeneity of the study population, these analyses were intended to assess the consistency of the observed ARIE pattern across major baseline, clinical, and treatment-context subgroups rather than to support formal subgroup inference. Treatment-context categories were defined according to exposure status and were not necessarily mutually exclusive.

Abbreviation: ECOG, Eastern Cooperative Oncology Group; IMRT, intensity-modulated radiation therapy; VMAT, volumetric modulated arc therapy; NSCLC, non-small cell lung cancer; SCLC, small cell lung cancer; CRT, chemo-radiation therapy; CIRT, chemo-immuno-radiation therapy.

Supplementary Table 3. Acute toxicities in the per-protocol population (n=69).

Toxic events (RTOG)	Grade 1, n (%)	Grade 2, n (%)	Grade 3, n (%)	Grade 4, n (%)	Grade 5, n (%)	Total events, n (%)
**Esophagitis**	12 (17.4%)	5 (7.2%)	1 (1.4%)	0 (0%)	No Grade 5 toxic events happened.	18 (26.1%)
**Pneumonitis** [a]	12 (17.4%)	10 (14.5%)	2 (2.9%)	0 (0%)		24 (34.8%)
**Upper-GI injury** [b]	14 (20.3%)	3 (4.3%)	2 (2.9%)	0 (0%)		19 (27.5%)
**Hematologic toxicity** [c]	22 (31.9%)	26 (37.7%)	5 (7.2%)	1 (1.4%)		54 (78.3%)

Abbreviation: RTOG, Radiation Therapy Oncology Group; Upper-GI, upper gastrointestinal.
[a] pneumonitis caused by radiotherapy and/or concurrent immunotherapy; [b] upper-GI injury caused by radiotherapy and/or concurrent chemotherapy and/or concurrent immunotherapy; [c] hematologic toxicity caused by radiotherapy and/or concurrent chemotherapy and/or concurrent immunotherapy, including leukopenia, neutropenia, thrombocytopenia, and anemia.

Supplementary Table 4. Characteristics of the historical cohort (n=51) and the treatment

cohort (n=80).

Clinical features	No. of patients (%)	
	historical cohort	treatment cohort
**Age (median [range]), years**	64 (60 – 66.5)	68 (48 - 80)
≥ 60	40 (78.4%)	70 (87.5%)
<60	11 (21.6%)	10 (12.5%)
**Gender**		
Female	11 (21.6%)	14 (17.5%)
Male	40 (78.4%)	66 (82.5%)
**Clinical AJCC stage (8th edition)**		
II	4 (7.8%)	5 (6.26%)
IIIA	13 (25.5%)	15 (18.75%)
IIIB	18 (35.3%)	19 (23.75%)
IIIC	10 (19.6%)	17 (21.25%)
IV	6 (11.8%)	24 (30.0%)
**ECOG performance status**		
0~1	48 (90.0%)	72 (90.0%)
2	3 (10.0%)	8 (10.0%)
**Smoking history**		
Yes	38 (74.5%)	56 (70.0%)
No	13 (25.5%)	24 (30.0%)
**Comorbidities**		
Yes	31 (60.8%)	51 (63.75%)
No	20 (39.2%)	29 (36.25%)
**Histologic type**		
NSCLC	30 (58.8%)	33 (41.25%)
SCLC	21 (41.2%)	47 (58.75%)
**Tumor laterality**		
Left and mediastinal	19 (37.3%)	40 (50.0%)
Right and mediastinal	25 (49.0%)	32 (40.0%)
Mediastinal	7 (13.7%)	8 (10.0%)
**Chemotherapy**		
Concurrent chemotherapy	25 (49.0%)	49 (61.25%)
Sequential chemotherapy	21 (41.2%)	28 (35.0%)
No chemotherapy	5 (9.8%)	3 (3.75%)
**Concurrent immunotherapy**		
Yes	31 (60.8%)	47 (58.75%)
No	20 (39.2%)	33 (41.25%)
**Prescribed dose**		
45 Gy in 15 fractions, QD	4 (7.8%)	24 (30.0%)
45 Gy in 30 fractions, BID	13 (25.5%)	24 (30.0%)
50 Gy in 2Gy/fraction, QD	17 (33.4%)	15 (18.75%)
56 Gy in 2Gy/fraction, QD	5 (9.8%)	1 (1.25%)
60 Gy in 2Gy/fraction, QD	12 (23.5%)	16 (20.0%)
**Technology type, n (%)**		
IMRT	36 (70.6%)	72 (90.0%)
VMAT	15 (29.4%)	8 (10.0%)
**CTV (median [range]), cm^3**	104.4 (79.1 – 152.8)	96.5 (1.7 – 376.5)
**PTV (median [range]), cm^3**	227.6 (176.7 – 300.2)	214.9 (17.2 – 655.8)
**$D_{shortest}$ (median [IQR]), mm**	0 (0 – 0)	0 (0 – 0)
**Total Esophagus**		
Dmax (median [IQR]), cGy	5350.3 (5007.0 – 6139.7)	5269.5 (4794.7 – 5430.2)
Dmean (median [IQR]), cGy	2000.8 (1574.3 – 2358.1)	1471.8 (1147.5 – 1942.3)

Supplementary Table 5. Exploratory assessment of potential factors related to ARIE.

Variable	Comparison	HR	95%CI	*P*-value	adj-*P*-value	PHtest- *P* value
			**Part I: clinical factors**			
Age	per 1 year increase	0.98	0.93-1.03	0.400		0.783
Gender	Female vs. Male	1.24	0.46-3.31	0.672		0.518
Performance status	ECOG 2 vs. ECOG 0~1	0.83	0.20-3.53	0.802		0.390
Smoking history	Yes vs. No	1.09	0.45-2.64	0.840		0.346
Comorbidities	Yes vs. No	0.77	0.34-1.74	0.532		0.719
Histologic type	SCLC vs. NSCLC	1.28	0.56-2.93	0.555		0.150
Concurrent chemotherapy	Yes vs. No	1.33	0.57-3.11	0.510	0.765	0.878
Concurrent immunotherapy	Yes vs. No	2.34	0.93-5.89	0.072		0.630
Concurrent CIRT	Yes vs. No	2.46	1.10-5.51	0.028		0.892
			**Part II: geometric factors**			
$P_{Length\text{-}IEE/TE}$	per 5% increase	1.13	0.98-1.31	0.098		0.655
$P_{Volume\text{-}ES/TE}$	per 5% increase	1.28	1.08-1.52	0.004		0.646
$P_{Volume\text{-}IEE/TE}$	per 5% increase	1.07	0.94-1.22	0.308		0.833
			**Part III: radiation therapy related factors**			
Pass the ES-plan	Yes vs. No	0.37	0.15-0.93	0.035		0.856
Fractionation	1.5Gy/Fx vs. others	0.57	0.21-1.53	0.268	0.268	0.801
	2Gy/Fx vs. others	0.52	0.22-1.26	0.151	0.226	0.037
	3Gy/Fx vs. others	3.09	1.38-6.88	0.006	0.018	0.113
BED range	[50, 60) vs. others	1.91	0.79-4.60	0.151	0.226	0.037
	[60, 70) vs. others	0.14	0.02-1.07	0.058	0.174	0.534
	[70, Inf) vs. others	1.27	0.50-3.19	0.616	0.616	0.040
			**Part IV: dosimetric factors**			
**Esophagus-Sparing Region**						
Dmax	per 1Gy increase	1.04	0.99-1.10	0.145		0.105
Dmean	per 1Gy increase	1.00	0.95-1.05	0.923		0.581
$V20_{Gy}$	per 5% increase	0.99	0.90-1.09	0.822		0.808
$V30_{Gy}$	per 5% increase	1.01	0.92-1.12	0.847		0.733
$V40_{Gy}$	per 5% increase	1.05	0.94-1.17	0.373		0.719
$V50_{Gy}$	per 5% increase	1.13	0.96-1.33	0.157		0.386
$V60_{Gy}$	per 5% increase	1.61	1.06-2.45	0.027		0.043
**Total Esophagus**						
Dmax	per 1Gy increase	1.02	0.97-1.08	0.463		0.086
Dmean	per 1Gy increase	1.06	1.01-1.12	0.037		0.649
$V20_{Gy}$	per 5% increase	1.13	0.98-1.30	0.089		0.899
$V30_{Gy}$	per 5% increase	1.12	0.98-1.28	0.087		0.992
$V40_{Gy}$	per 5% increase	1.13	0.99-1.29	0.063		0.968
$V50_{Gy}$	per 5% increase	1.15	0.96-1.38	0.142		0.388
$V60_{Gy}$	per 5% increase	1.23	0.91-1.68	0.184		0.167

Abbreviations: PHtest, proportional hazards assumption test; ECOG, Eastern Cooperative Oncology Group; SCLC, small cell lung cancer; NSCLC, non-small cell lung cancer; CIRT, chemo-immuno-radiation therapy; P_{Length}, the percentage of length; IEE, injury-effective esophagus; TE, total esophagus; P_{Volume}, the percentage of volume; ES, esophagus-sparing; CI*, concordance index; n Gy/Fx, a single fractionated dose of n Gy; BED, biologically effective dose;Inf, infinitive; Dmax, max dose; Dmean, mean dose.

Supplementary Table 6. Comparison of studies about Esophagus-sparing techniques.

	Al-Halabi et al [21]	Kamran et al [22]	Lim et al [23]	Wang et al [15]	Ma et al [16]	Granton et al [17]	Louie et al [18]	Zhou et al [19]	Zhou et al [20]	This study
**Focus Area**	CE			rapid dose falloff						Segmental CE
**Patients**	20 pts, NSCLC	25 pts, NSCLC	159 pts, NSCLC	30 pts, SCLC	87 pts, NSCLC	15 pts	90 pts	75 pts, NSCLC	18 pts	80 pts
**Treatment**	dCCRT	dCCRT, with/without durvalumab	dCCRT, half durvalumab 41 pts CES-applied	dCCRT	dCCRT, 44 pts ES-applied,	Palliative Radiation	Palliative Radiation, 45 pts ES-applied	Hypo CCRT + hypo-Boost	Hypo CCRT + hypo-Boost, ICI in selected pts	dCCRT, half immunotherapy
**RT Technique**	IMRT	IMRT	IMRT	IMRT/VMAT	IMRT	VMAT	IMRT/VMAT	IMRT/VMAT	IMRT	IMRT/VMAT
**Prescribed RT dose**	At least 63 Gy	70Gy/35Fx	66Gy/30Fx	45Gy/15Fx	SIB, median 65Gy	30Gy/10Fx	30Gy/10Fx<br>20Gy/5Fx	40Gy/10Fx + 24-28Gy/6-7Fx	①60Gy/20Fx<br>②60Gy/15Fx<br>③60Gy/12Fx	BED ≥ 51.75 Gy
**Distance**	GTV within 1cm of Esophagus		ITV within 1cm of Esophagus	CTV within 1cm of Esophagus	GTV within 32mm of Esophagus	5 cm of the esophagus within the POP treatment field		Not mentioned		CTV within 5mm of Esophagus
**Dose constraints**	①CE Dmax ≤ 55 Gy<br>②CE $V45_{Gy}$ as low as possible	①CE Dmax ≤ 60 Gy<br>②CE $V55_{Gy}$ < 0.55 cc<br>③CE $V45_{Gy}$ < 2.5 cc<br>Deviations are permitted	①CE Dmax ≤ 60 Gy<br>②CE $V55_{Gy}$ < 0.55 cc<br>③CE $V45_{Gy}$ < 2.5 cc<br>Un-/partly/ fully satisfied	①TE Dmax ≤ 50 Gy<br>②TE Dmean ≤ 34 Gy<br>③AE < 49.5 Gy<br>④AE1 < 47.25 Gy<br>⑤AE2 < 42.75 Gy<br>⑥AE3 < 40.50 Gy<br>⑦AE4 < 36.00 Gy<br>⑧AE5 < 22.50 Gy	①a 3mm margin been added to Esophagus to allow dose decline<br>②TE Dmax < 65Gy<br>③TE $V50_{Gy}$ < 30%	①A 5mm ring been created around the Esophagus to allow dose falloff<br>②TE Dmax < 80% PD		①a 3mm margin been added to Esophagus to allow dose decline<br>②smaller PTV-GTV margin for lesions close to esophagus<br>③TE-C1 Dmax≤ 34 Gy<br>④TE-C2 Dmax≤ 24 Gy	**DL1/DL2/DL3**<br>TE-C1 Dmax< 45Gy/36Gy/25Gy,<br>TE-C1 V30< 30%/20%/ V20<20%,<br>TE-C2 Dmax< 16Gy/18Gy/25Gy,<br>TE-C2 V10/V10/V20<20 %	$Pv = \frac{Esophagus\ within\ PTV}{Esophagus\ in\ same\ secti}$<br>① Pv < 1/3, ES Dmean = 1/3 PD<br>② 1/3 ≤ Pv < 2/3, ES Dmean = 1/2 PD<br>③ Pv >2/3, ES Dmean = 2/3 PD
**Actual RT Dose**	70.2 Gy (median)	68Gy/34Fx (1pt), 70Gy/35Fx	66Gy/30Fx	42Gy/14Fx (1pt), 45Gy/15Fx	ES-applied: 63.8 Gy	30Gy/10Fx	30Gy/10Fx, 20Gy/5Fx (36pts)	64 Gy (median)	60Gy	BED 58.5 Gy (median)

Supplementary Table 5. Comparison of studies about Esophagus-sparing techniques. (*continued*)

	Al-Halabi et al [21]	Kamran et al [22]	Lim et al [23]	Wang et al [15]	Ma et al [16]	Granton et al [17]	Louie et al [18]	Zhou et al [19]	Zhou et al [20]	This study
**ARIE incidence**	G1: 55%<br>G2: 20%<br>G3: 0%<br>G4-5: 0%	G1: not mentioned<br>G2: 24-28%<br>G3: 0%<br>G4-5: 0%	CES-applied: 53.7% pts use Pain Killer<br>39.0% pts with AQA score 2-3	G0-1: 76.7%<br>G2: 20%<br>G3: 3.3%<br>G4-5: 0%	ES-applied:<br>G1: 40.9%<br>G2: 45.5%<br>G3: 4.5%<br>G4-5: 0%	LBK-NTCP model:<br>2%	G1: not mentioned<br>G2+: 2%<br>higher 2-week ECS score	G1: 20%<br>G2: 26.7%<br>G3: 5.3%<br>G4-5: 0%	G1: 33.3%<br>G2: 27.7%<br>G3-5: 0%	G1: 18.75%<br>G2: 10.0%<br>G3: 1.25%<br>G4-5: 0%
**Esophagus dosimetrics**	CE Dmax 62.3 Gy<br>CE $V60_{Gy}$ 0.5%<br>CE $V55_{Gy}$ 0.4 cc<br>CE $V45_{Gy}$ 2.1 cc<br>TE Dmax 67.5 Gy<br>TE Dmean 23.1 Gy<br>TE $V60_{Gy}$ 5.3%	CE Dmax 66 Gy<br>CE $V55_{Gy}$ 1.4 cm^3<br>CE $V45_{Gy}$ 2.7 cm^3<br>TE Not mentioned	CES-applied:<br>CE Not mentioned<br>TE $V60_{Gy}$ 1.1 cc<br>TE $V40_{Gy}$ 5.2 cc	TE Dmax 47.38 Gy<br>TE Dmean 13.52 Gy<br>AE1 Dmax 41.18 Gy<br>AE2 Dmax 34.24 Gy<br>AE3 Dmax 29.04 Gy<br>AE4 Dmax 18.62 Gy<br>AE5 Dmax 4.96 Gy	ES-applied:<br>TE Dmax 64.67 Gy<br>TE Dmean 26.25 Gy<br>TE $V45_{Gy}$ 33.76%<br>TE $V50_{Gy}$ 20.75%<br>TE $V55_{Gy}$ 11.25%<br>TE $V60_{Gy}$ 6.12%	TE Dmean 8 Gy	TE Dmax 23.8 Gy<br>TE Dmean 9.8 Gy	TE Dmax 61.72Gy<br>TE Dmean 27.67Gy	TE-Total Dmax 56.6Gy<br>TE-DL1 Dmax 62.1Gy<br>TE-DL2 Dmax 56.7Gy<br>TE-DL3 Dmax 52.6Gy	ES Dmax 50.8 Gy<br>ES Dmean 22.5 Gy<br>ES $V40_{Gy}$ 19.3%<br>ES $V50_{Gy}$ 4.1%<br>ES $V60_{Gy}$ 0.7%<br>TE Dmax 53.2 Gy<br>TE Dmean 15.8 Gy<br>TE $V40_{Gy}$ 17.5%<br>TE $V50_{Gy}$ 6.8%<br>TE $V60_{Gy}$ 2.0%

Abbreviations: CE, contralateral esophagus; pts, patients; NSCLC, non-small cell lung cancer; SCLC, small cell lung cancer; dCCRT, definitive concurrent chemoradiotherapy; CES, contralateral esophagus-sparing; ES, esophagus-sparing; RT, radiation therapy; IMRT, intensity modulated radiation therapy; VMAT, volumetric modulated arc therapy; SIB, simultaneous integrated boost; BED, biologically effective dose; GTV, gross tumor volume; CTV, clinical target volume; ITV, internal target volume; POP, parallel-opposed pair; TE, total esophagus; AE, abutting esophagus; Gx(0-5), grade 0-5; AQA, analgesic quantification algorithm; LKB-NTCP, Lyman-Kutcher-Burman normal tissue complication probabilities; ECS, esophageal cancer subscale.
